# Hedgehog/Gli1 signaling pathway regulates MGMT expression and chemoresistance to temozolomide in human glioblastoma

**DOI:** 10.1186/s12935-017-0491-x

**Published:** 2017-12-04

**Authors:** Ke Wang, Dongjiang Chen, Zhouqi Qian, Daming Cui, Liang Gao, Meiqing Lou

**Affiliations:** Neurosurgical Department, Shanghai Tenth People’s Hospital, Tongji University, 301 Middle Yanchang Road, Shanghai, 200072 China

**Keywords:** Glioblastoma, Chemoresistance, MGMT, Temozolomide, Hedgehog signaling pathway, Gli1

## Abstract

**Background:**

Chemoresistance of glioblastoma (GBM) is a feature of this devastating disease. This study is to determine the relationship between Hedgehog (HH)/Gli1 signaling pathway and chemoresistance to temozolomide (TMZ) in human GBM.

**Methods:**

We analyzed Gli1 nuclear staining and O^6^-methylguanine DNA methyltransferase (MGMT) expression in 48 cases of primary GBM tissues by immunohistochemistry. Quantitative PCR, western blot, methylation-specific PCR, cell proliferation and apoptosis assay were used to investigate changes of MGMT expression and chemosensitivity to TMZ after manipulating HH/Gli1 signaling activity in A172 and U251 GBM cell lines. Chromatin immunoprecipitation assay was utilized to identify potential Gli1 potential binding sites in MGMT gene promoter region. We established GBM xenografts using U251 cells to assess whether inhibiting HH/Gli1 signaling activity restored chemosensitivity to TMZ.

**Results:**

O^6^-Methylguanine DNA methyltransferase-positive GBM tissues had a significantly higher rate of Gli1 nuclear staining than MGMT-negative ones (67.7% vs. 32.3%, p = 0.0159). Activation of HH/Gli1 signaling by pcDNA3.1-Gli1 cell transfection in A172 cells led to increased MGMT expression and enhanced resistance to TMZ treatment. Inhibition of the HH/Gli1 signaling by cyclopamine in U251 cells resulted in decreased MGMT expression and increased sensitivity to TMZ treatment. Both ways altered MGMT levels without changing the MGMT promoter methylation. The potential binding site of Gli1 in the MGMT gene promoter region was located at – 411 to − 403 bp upstream the transcriptional start site. The in vivo study revealed a synergistic effect on tumor growth inhibition with the combined administration of cyclopamine and TMZ.

**Conclusions:**

This study shows that HH/Gli1 signaling pathway regulates MGMT expression and chemoresistance to TMZ in human GBM independent from MGMT promoter methylation status, which offers a potential target to restore chemosensitivity to TMZ in a fraction of GBM with high MGMT expression.

## Introduction

Glioblastoma (GBM) is the most common and devastating tumor in the central nervous system. Despite optimal treatment, the patients with this disease can expect a dismal prognosis, a mean survival of 12–15 months after initial diagnosis [1]. Chemotherapy plays an important role in the treatment of the disease. Currently, temozolomide (TMZ)-based chemotherapy significantly improves prognosis, and is recommended as a standard care for GBM patients [2]. The main effect of TMZ is to methylate the O^6^ residues of guanine so as to prevent DNA duplication during cell proliferation and to induce cell death and apoptosis [3]. However, a DNA repair enzyme, O^6^-methylguanine DNA methyltransferase (MGMT), is able to reverse the anti-tumor effect of TMZ, and mainly contributes to the chemoresistance in a fraction of patients. Normally, high promoter methylation status which means low MGMT activity predicts good response to TMZ chemotherapy and results in a longer survival period in GBM patients while low promoter methylation status, which leads to high MGMT expression is linked to a remarkable chemoresistance and a shorter survival period [4, 5]. Bases on previous studies, there are about 50% patients with high MGMT activity in primary GBM [2], and an even higher percentage in recurrent ones [6]. Therefore, it is of great significance to alleviate the chemoresistance of GBM with high MGMT expression.

Hedgehog (HH) signaling pathway plays an important role in the development of central nervous system during embryogenesis [7, 8]. In mammals, the canonical HH signaling pathway starts with binding of one of the three extracellular ligands (Sonic HH, Indian HH and Desert HH) to the transmembrane receptor protein Patched (PTCH). Two PTCH receptors, PTCH1 and PTCH2, have been identified for the ligands. Binding of HH ligands to PTCH relieves another transmembrane protein, smoothened (SMO), from the inhibitory effect of PTCH, resulting in transcription of downstream target genes through the Gli transcription factors [9]. The Gli protein family, which consists of Gli1, Gli2 and Gli3, serves as the mediators of the HH signaling pathway. Gli1 acts as a main transcriptional activator in HH signaling pathway [10]. Aberrant activation of HH/Gli1 signaling pathway has been implicated in a fraction of GBM, and inhibiting HH/Gli1 signaling results in tumor growth suppression [11]. Our previous study showed that Gli1 was a potential target to alleviate multidrug resistance of human glioma by regulating the transcription of a series of chemoresistance-associated genes [12], but the relationship between HH/Gli1 signaling and MGMT expression in GBM is yet to be clarified.

In this study, we first evaluated the relationship between Gli1 activity and MGMT expression in primary GBM tissues. In vitro cultured GBM cells lines, we explored the effects and mechanism of regulating the HH/Gli1 signaling pathway on MGMT expression and chemoresistance to TMZ. Finally, we performed in vivo studies to verify whether the signaling pathway can serve as a potential target to overcome chemoresistance in GBM with high MGMT activity.

## Methods

### Immunohistochemistry

With the approval of the institutional ethics committee, forty-eight patients with primary GBM whom underwent tumor resection in the Neurosurgical Department of Shanghai Tenth People’s Hospital from Jan 2011 to Dec 2013 were given informed consent and enrolled in this study. The median age was 58 years (range, 40–75); 20 patients were male and 28 female. All patients received surgical treatment and specimens were fixed with 10% formalin, embedded in paraffin, and examined histopathologically. Immunohistochemistry was done as previously described [13], and the specimens were not affected by the chemotherapy. Primary antibodies were used as follow: rabbit polyclonal anti-Gli1 antibody (1:100, ab49314, Abcam) and mouse monoclonal anti-MGMT antibody (1:100, ab39253, Abcam). The percentage of tumor cells with Gli1 nuclear staining to total cancer cells being > 10%, it was judged to be Gli1-positive. With the ratio of tumor cells stained with MGMT > 5%, it was judged to be MGMT-positive [14]. Sections from the same tissues without application of the primary antibodies were used as negative controls.

### Cell culture, reagents and immunofluorescence

The GBM cell lines, U87, A172, and U251, were purchased from the Cell Bank of the Chinese Academy of Sciences (Shanghai, China). The cells were regularly verified for cell morphology and growth characteristics by microscopic analysis. All the cells were cultured in 10% FBS-supplemented DMEM and maintained at 37 °C in a humidified atmosphere of 5% CO_2_ and 95% air. The procedures to obtain and stain glioma stem/progenitor (GSP) cells from U87 cells were as previously described [15]. The growth factor EGF and bEGF were purchased from PeproTech; B27 was purchased from Gibco. Rabbit polyclonal anti-Gli1 antibody (1:100, ab49314, Abcam) and mouse monoclonal anti-MGMT antibody (1:100, ab39253, Abcam) were used to stain GSP cells for immunofluorescence assay. Slides were stained with DAPI (Beyotime Biotechnology) for 5 min prior to examination using a confocal microscope.

### Western Blot analysis

Whole-cell extracts were prepared after lysis in buffer containing RIPA (Beyotime Biotechnology) and PMSF (Beyotime Biotechnology) (100:1). Equal amounts of protein (30 μg/sample) were separated by 10 or 5% SDS-PAGE gels. Proteins were transferred to polyvinylidene difluoride membranes and then blocked in 5% BSA dissolved in PBST for 1 h. Membranes were incubated with primary antibodies (Gli1: GTX124274, GeneTex; MGMT: SAB1406122, Sigma-Aldrich; β-actin: ab8226, Abcam) in 5% BSA at 4 °C overnight followed by secondary antibody for 1 h at room temperature. Bands were visualized on an Odyssey Infrared Imaging System (LI-COR Biosciences).

### Real-time quantitative PCR and methylation-specific PCR

Total RNA was extracted from cells using Trizol reagent (Invitrogen). To detect the mRNAs of the different genes examined, the primers applied for real-time PCR are as follows: Gli1 forward: 5′-TTCCTACCAGAGTCCCAAGT-3′; reverse: 5′-CCCTATGTGAAGCCCTATTT-3′; MGMT forward: 5′-CCTGGCTGAATGCCTATTTC-3′; reverse: 5′-GATGAGGATGGGGACAGGATT and GAPDH forward: 5′-TGCACCACCAACTGCTTAGC-3′; reverse: 5′-GGCATGGACTGTGGTCATGAG-3′. Average level of GAPDH RNA was used as internal control. Genomic DNA was extracted and treated with sodium bisulfite for methylation-specific PCR (MSP) using the EpiTect Plus LyseAll Lysis, Plus DNA Bisulfite, MSP Kit (Qiagen). The primers used for methylated product were: MGMT-M forward: 5′-GTTTCGGGTTTAGCGTAGTC-3′; reverse: 5′-TATCACAAAAATAATCCGCG-3′. The unmethylated primers are: MGMT-U forward: 5′-GTTTTGGGTTTAGTGTAGTT-3′; reverse: 5′-TATCACAAAAATAATCCACA.

### Construction of Gli1 expression vector

The RT-PCR amplification mixture contained the primers followed: Gli1 forward: AGGGAGACCCAAGCTGgctagcATGTTCAACTCGATGACCCCACCACCAA; reverse: TAAGCTTGGTACCTCAtctagaTTAGGCACTAGAGTTGAGGAATTCTG. GCTAGC and TCTAGA were recognition sequences of the restriction endonucleases, *Nhe*I and *Xba*I.

### Cell transfection

Cells were transiently transfected using Lipofectamine 2000 (Invitrogen) with vector pcDNA3.1-Gli1 or the empty vector pcDNA3.1. Cells were used at 24, 48, 72, 96 and 120 after transfection.

### Cells proliferation and apoptosis assay

Cells were plated in 96-well plates at a density of 1 × 10^3^ cells per well and incubated in 10% FBS-supplemented DMEM with 100 μM TMZ (Enzo Life Sciences) and maintained at 37 °C under a humidified atmosphere of 5% CO_2_ and 95% air for 120 h. Cyclopamine (Cayman chemical) at the concentration of 5 μM was added as indicated in the “Results” section. Cell proliferation was detected by the cell counting kit-8 (CCK8, Dojindo) and the absorbance was measured by microplate reader at 450 nm.

Cells were cultured in 6-well plates at a density of 2 × 10^5^ cells per well for the apoptosis assay. The apoptotic effects of treatments were determined by using Vybrant Apoptosis Assay Kit (Invitrogen), followed by fluorescence-activated cell sorter analysis using a FACScanto flow cytometry.

### Chromatin Immunoprecipitation (ChIP) analysis

The ChIP analysis was conducted in the U251 cell line, using the EpQuik™ Chromatin Immunoprecipitation Kit (p-2002-2, epigentek) according to the manufacturer’s instructions. 1 μg mouse IgG and 1 μg anti-RNA polymerase II served as a negative and positive control respectively. For immunoprecipitation, 2 μg antibody against Gli1 (NB600-600, Novus Biologocals) was added to each well. The primers applied for ChIP analysis were as follows: Homo MGMT1 for site 1 (169 bp): forward: 5′-CCCCATCTCCAAATAAGGTC-3′; reverse: 5′-TAGACACTGCCAGAGCCTGA-3′; Homo MGMT2 for site 2 (210 bp): forward: 5′-GACGGCATCGCCCACCACA-3′; reverse: 5′-GCCCGAGTGGTCCTGAAAGC-3′; Homo MGMT3 for site 3 (276 bp): forward: 5′-TCAGGCGGAAGCTGGGAAGG-3′; reverse: 5′-CCGAGGACCTGAGAAAAGCAAGAG-3′. Primers for RegIV: forward: 5′-CTCGGAAGGTTTCTAATC-3′; reverse: 5′-TTCAACATGCGTGAGTTT-3′.

### In vivo study

The Committee on Ethical Use of Animals of Shanghai Tenth People’s Hospital approved the in vivo study. The animal studies complied with the ARRIVE guidelines. Female nude mice of 4 weeks old were obtained from the Changzhou Cavens Laboratory Animal Co. Ltd. U251 cells in log-phase growth were suspended in PBS at 5 × 10^7^/mL and then subcutaneously injected 200 μL per mouse into the right back of nude mice. When the tumor diameter reached about 5 mm, the mice were randomly divided into four groups with 5 mice per group and treated by the following agents: (a) the control: ethanol (1 mL/kg, days 1–2, p.o.) plus DMSO (1 mL/kg, days 1–5, p.o.); (b) TMZ alone: TMZ dissolved in equivalent dose of DMSO (42 mg/kg, days 1–5, p.o.) plus ethanol (1 mL/kg, days 1–2, p.o.); (c) cyclopamine alone: cyclopamine dissolved in equivalent dose of ethanol (25 mg/kg, days 1–2, p.o.) plus DMSO (1 mL/kg, days 1–5, p.o.); (d) TMZ plus cyclopamine: TMZ plus cyclopamine at the equivalent doses. Mouse body weight and the tumor size were measured every 5 days. The approximate volume of the tumor was calculated using the formula (length × width^2^)/2. All mice were sacrificed at day 30 and tumor tissues was stained by immunohistochemistry for Gli1 and MGMT expression.

### Statistical analysis

All statistical analyses were performed with SPSS 16.0 for windows. Data was presented as the mean ± SD, and were compared by two-tailed unpaired Student’s *t* test or Person Chi Square test. Difference was considered significant when *p* value was below 0.05.

## Results

### Gli1 activity correlates with MGMT expression in primary GBM tissues

**Fig. 1:**
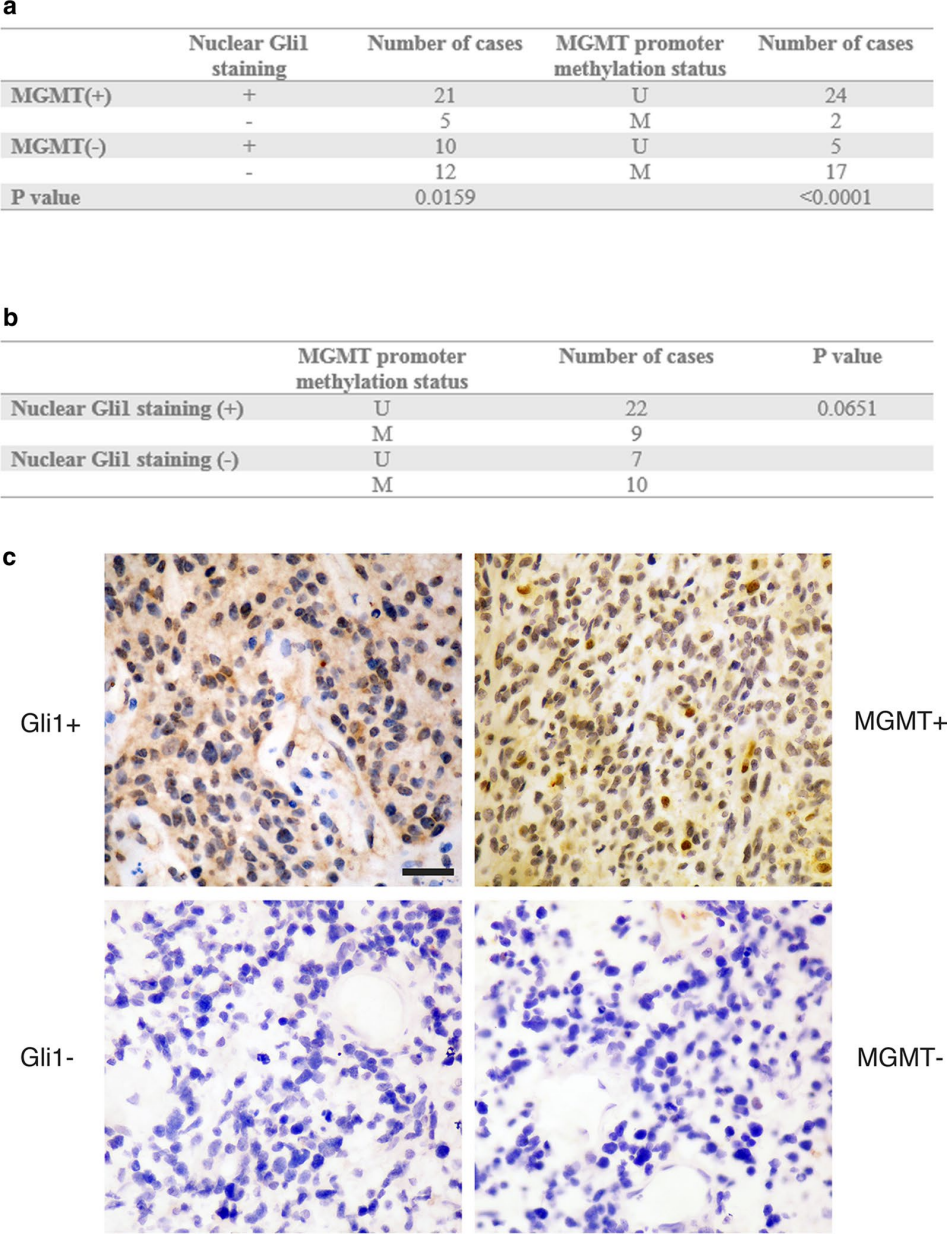
Relationship between MGMT expression, Gli1 nuclear staining and MGMT promoter methylation status in 48 cases of surgically-resected primary GBM tissues (**a**, **b**). Representative photomicrographs showed two GBM cases with distinct Gli1 activity (**c**). The top two photos represent a Gli1-positive GBM with visible nuclear staining of Gli1 (left) and the same sample stained with MGMT antibody showed that the percentage of MGMT staining was far more than 5% (right). The bottom two photos represent a Gli1-negative case with invisible nuclear staining of Gli1 (left). The same sample stained with MGMT antibody showed the ratio of MGMT staining was less than 5% (200×). The scale bar on the top left panel applies to the rest of the photographs and indicates 20 μm

As nuclear staining of Gli1 is a reliable marker of HH pathway activity in brain glioma [16], the percentage of Gli1 nuclear staining was used to divide the 48 surgically resected GBM samples into Gli1-positive or Gli1-negative groups. Only two patients classified as IDH-mutant and both patients belonged to the MGMT promoter methylated and MGMT, Gli1 negative group. As noted in the Fig. 1a, 31 cases and 26 cases were respectively judged to be Gli1-positive and MGMT-positive. 29 out of the 48 patients were MGMT promoter unmethylated status. The percentage of Gli1 nuclear staining in MGMT-positive tissues was significantly higher than that in MGMT-negative ones (67.7% vs. 32.3%, p = 0.0159). That suggests a positive correlation between HH/Gli1 pathway activity and MGMT expression (Fig. 1c). Unsurprisingly, the MGMT promoter methylation status showed a stronger correlation with MGMT level (p < 0.0001). However, when comparing Gli1 expression with MGMT promoter status, there was no statistical significance (p = 0.0651, Fig. 1b). Combined together, the data showed that both Gli1 and MGMT promoter status have relevance with MGMT activity. Although the MGMT promoter occupied a dominant position here, Gli1 potentially plays an independent role.

### Expression of Gli1 and MGMT in GBM cell lines

**Fig. 2:**
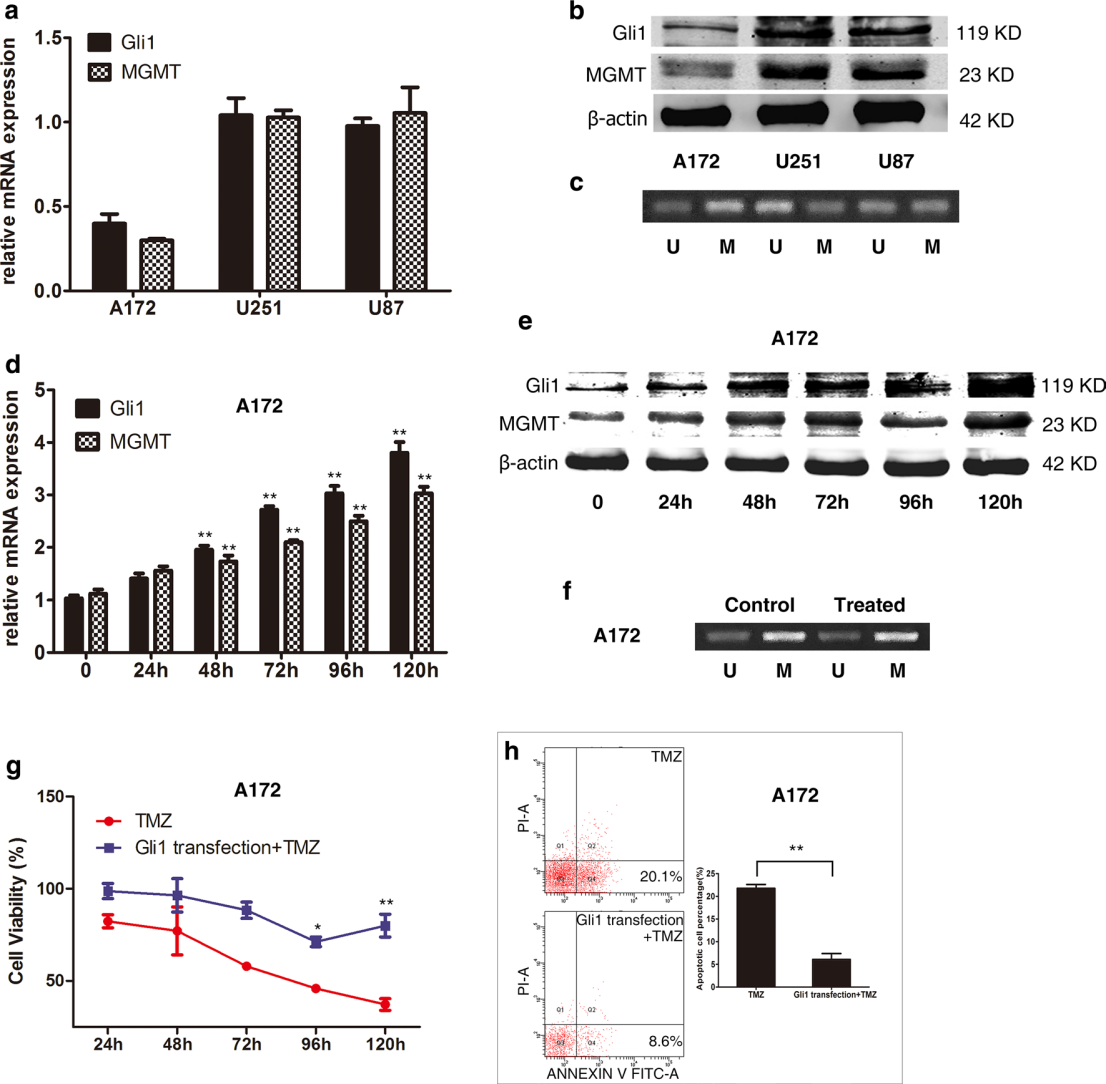
Measurement of Gli1 and MGMT expression by real-time quantitative PCR (**a**) and western blot analysis (**b**) in the three GBM lines, including A172, U251, and U87. The expression of the ubiquitously expressed β-actin gene was measured as the control. The MGMT promoter methylation status was also as showed (**c**). The lane labelled as ‘M’ means methylated; ‘U’ stands for unmethylated. Gli1 expression was increased by pcDNA3.1-Gli1 transfection in A172 cells measured at mRNA and protein levels since 48 h and thereafter (**d**–**f**), as well as MGMT expression and promoter status. When exposed to TMZ treatment, cell viability in the A172 cells transfected with pcDNA3.1-Gli1 was significantly higher than that in the control since 96 h and thereafter (**g**). The percentage of apoptotic cells at 120 h after pcDNA3.1-Gli1 transfection was significantly lower than in the control cells (**h**). *p < 0.05; **p < 0.01

To elucidate the relationship between HH/Gli1 signaling and MGMT expression, we analyzed mRNA and protein levels of Gli1 and MGMT in several commonly used GBM cell lines by real-time quantitative PCR and western blot analysis. All these three cell lines used were IDH-wildtype cell lines. As shown in Fig. 2a and b, the results revealed a consistent expression of Gli1 and MGMT in U87, U251, and A172 cell lines. The A172 cells had relative low levels of Gli1 and MGMT when compared with U251 and U87 cells. Moreover, we evaluated the MGMT promoter methylation status of these three cell lines by methylation-specific PCR (MSP, Fig. 2c). More methylated DNA was detected in U251 and more unmethylated DNA was generated by A172 while U87 got around 50%. Therefore, we used A172 cells for up-regulating the HH/Gli1 signaling activity and U251 cells for down-regulating the pathway in the following experiments.

### Gli1 overexpression by pcDNA3.1-Gli1 transfection induces MGMT expression and enhances chemoresistance to TMZ in A172 cells

To verify whether HH/Gli1 signaling pathway is involved in the regulation of MGMT expression in GBM cell lines, we used pcDNA3.1-Gli1 to increase the Gli1 expression in A172 cells. The mRNA and protein levels of Gli1 and MGMT were measured every 24 h until 120 h after cell transfection. Quantitative PCR measurements showed that the relative changes of Gli1 mRNA after transfection in A172 cells were 1.0 ± 0.1, 1.4 ± 0.2, 1.9 ± 0.1, 2.3 ± 1.0, 2.7 ± 0.1, 3.0 ± 0.2, and 3.8 ± 0.3 at the time points shown in Fig. 2d. The relative changes of MGMT mRNA were 1.1 ± 0.1, 1.6 ± 0.1, 1.7 ± 0.2, 2.1 ± 0.1, 2.5 ± 0.2, and 3.0 ± 0.2 at those time points, respectively. Both the mRNA levels of Gli1 and MGMT were significantly increased since 48 h after the transfection (p < 0.01). Western blot analysis also confirmed the consistent increase of Gli1 and MGMT protein levels after pcDNA3.1-Gli1 transfection in A172 (Fig. 2e). The MGMT promoter statement did not change when measured at 120 h post the treatment (Fig. 2f). This result verified our hypothesis that Gli1 can affect MGMT level without changing the promoter condition. Furthermore, we investigated whether Gli1 overexpression would influence chemosensitivity to TMZ. A172 cells transfected with pcDNA3.1-Gli1 or empty vector were exposed to TMZ at the concentration of 100 μM. Cell viability was analyzed at the time points indicated in the Fig. 2g. The results showed that cell viability in the A172 cells transfected with pcDNA3.1-Gli1 was significantly higher than those in the control cells at 96 and 120 h (p < 0.05, p < 0.01) respectively. The percentage of apoptotic cells at 120 h after pcDNA3.1-Gli1 transfection was significantly lower than in the control cells (6.1 ± 2.3% vs. 21.8 ± 1.5%, p < 0.001, Fig. 2h).

### Inhibiting HH/Gli1 signaling by cyclopamine reduces MGMT expression and decreases chemoresistance to TMZ in U251 cells

**Fig. 3:**
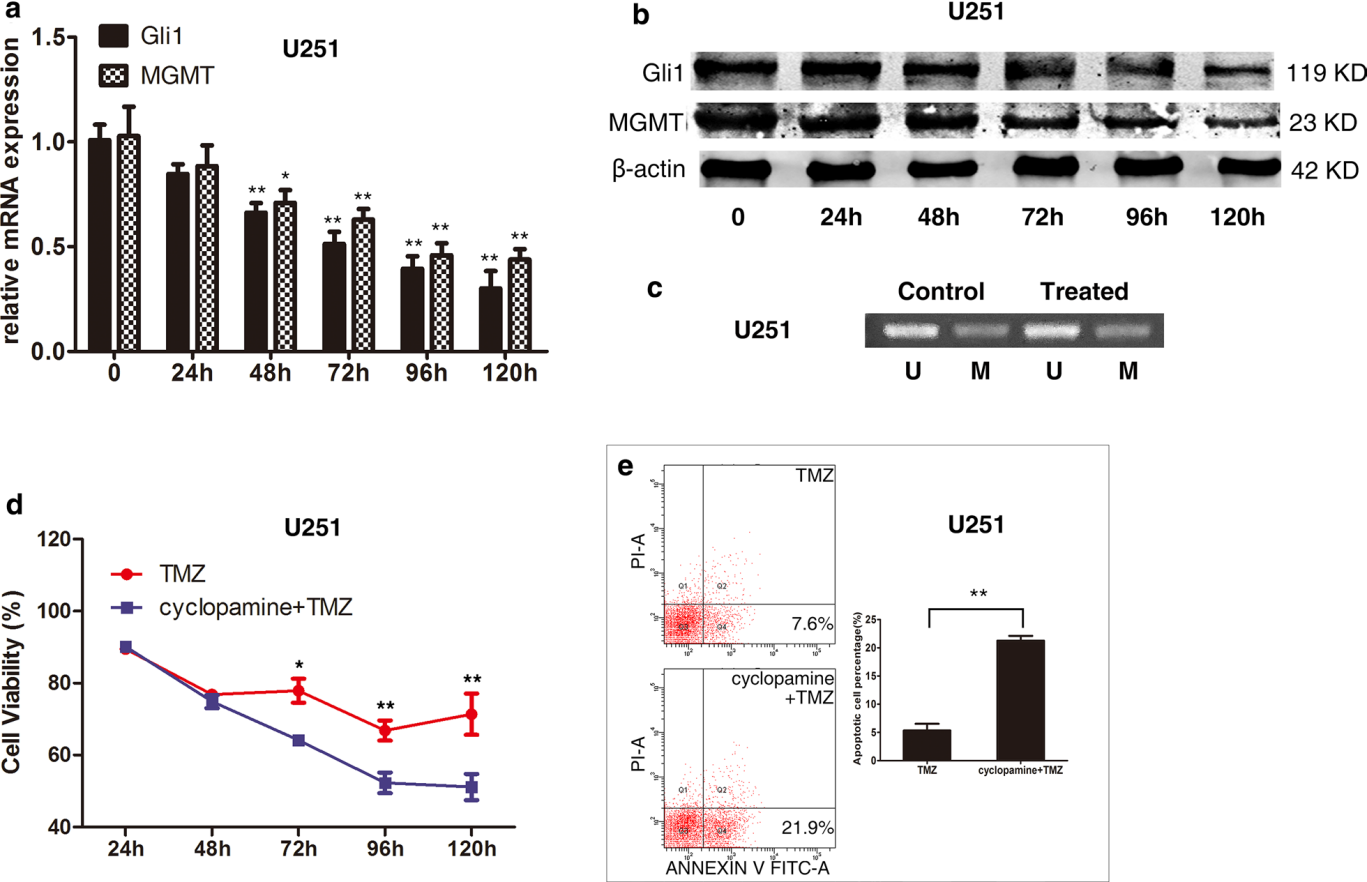
The mRNA and protein levels of Gli1 and MGMT in U251 cells were significantly inhibited 48 h after treated by 5 μM cyclopamine (**a** and **b**). The MGMT promoter statement remained unchanged like the A172 treatment (**c**). When exposed to TMZ, cell viability treated with cyclopamine was significantly lower than that in the control 72 h after the treatment (**d**). The percentage of apoptotic cells in U251 cells treated with cyclopamine was significantly higher than in the control at 120 h post-treatment (**e**)

We used cyclopamine, a commonly used HH pathway inhibitory drug targeting SMO, to block the HH/Gli1 signaling pathway in U251 cells. To produce a HH pathway-specific effect rather than a generally cytotoxic effect, the concentration of cyclopamine was used at 5 μM as previously discussed [17]. Quantitative PCR measurements showed that the relative Gli1 mRNA levels in U251 cells decreased to 1.0 ± 0.1, 0.8 ± 0.1, 0.7 ± 0.1, 0.5 ± 0.1, 0.4 ± 0.1, and 0.3 ± 0.1 at the time shown in the Fig. 3a. Meanwhile, the relative MGMT mRNA levels reduced to 1.0 ± 0.2, 0.9 ± 0.2, 0.7 ± 0.1, 0.6 ± 0.1, 0.5 ± 0.1, and 0.4 ± 0.1 at those time points. The mRNA levels of Gli1 and MGMT were significantly inhibited since 48 h after cyclopamine treatment (p < 0.01 and p < 0.05, respectively). Western blot analysis revealed that the MGMT protein levels decreased along with Gli1 expression (Fig. 3b). Same with the transfection, the alternate of the HH/Gli1 pathway could not affect the MGMT promoter methylation statement (Fig. 3c). Furthermore, we conducted cell proliferation and apoptosis assay using U251 cells exposed to 100 μM TMZ with or without 5 μM cyclopamine. The results showed that cyclopamine plus TMZ treatment reduced cell viability to 90.2 ± 2.1% at 24 h, 74.9 ± 3.2% at 48 h, 64.2 ± 1.5% at 72 h, 52.3 ± 5.0% at 96 h, and 51.1 ± 6.32% at 120 h when compared with vehicle while TMZ alone group was 89.5 ± 1.7%, 76.9 ± 1.3%, 77.9 ± 5.8%, 64 ± 5.4%, 71.4 ± 9.9%, demonstrating significant reduction at 72 h after the treatment (Fig. 3d). The percentage of apoptotic cells in the U251 cells treated by cyclopamine plus TMZ was significantly higher than that in TMZ alone at 120 h (21.3 ± 1.5% vs. 5.3 ± 2.0%, p < 0.001, Fig. 3e).

### Identification of potential Gli1 binding sites in the MGMT promoter

**Fig. 4:**
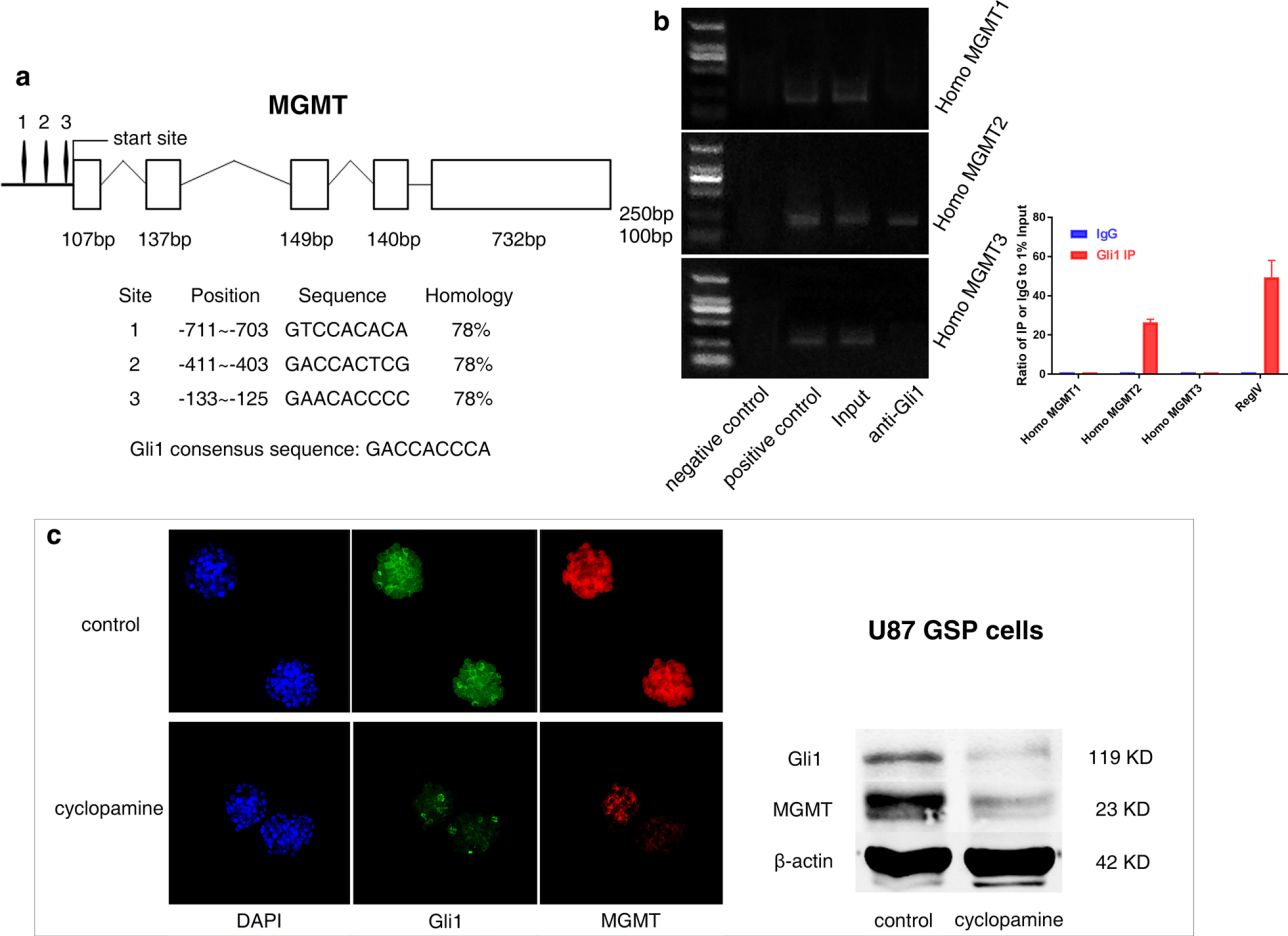
Location and sequence homology of the potential Gli1 binding sites (numbers 1–3) in relation to the structure of the MGMT gene. Blank frames represent the five exons and their length (**a**). The CHIP assay showed only the primers used for the Homo MGMT2 produced the DNA band in the anti-Gli1 lane. ChIP-qPCR also confirmed the result. Human RegIV promoter here served as a positive control for Gli1 binding site (**b**). Gli1 and MGMT expression were inhibited 72 h after treated by 5 μM cyclopamine in U87 GSP cells measured by immunofluorescence staining and western blot (**c**). Green (Gli1); red (MGMT); blue (DAPI)

The relationship between HH signaling pathway and MGMT expression prompted us to search the MGMT promoter for potential Gli1 binding sites to the DNA consensus sequence 5′-GACCACCCA-3′ [18]. Bioinformatics analysis was performed using BLAST and three potential binding sites were located within 1000 bp upstream of the MGMT gene transcriptional start site was identified. The homology of the three potential Gli1 binding sites was identically 78% (Fig. 4a). The results of agarose gel electrophoresis after DNA amplification demonstrated that only the primers used for Homo MGMT2 produced a positive DNA band in anti-Gli1 lanes. ChIP-qPCR shows the same result. Only Homo MGMT2 and RegIV primers, which served as a positive control, successfully got the amplification products (Fig. 4b). These findings indicates that the 5′-GACCACTCG-3′ sequence in the MGMT promoter is one of the potential Gli1-binding sites.

### Cyclopamine inhibits Gli1 and MGMT expression in the U87 GSP cells

We investigated Gli1 and MGMT expressions in the GSP cells cultured from U87 by immunofluorescence staining and western blot analysis, and the results showed measurable levels of both the genes before cyclopamine treatment. Both Gli1 and MGMT levels were markedly inhibited in the GSP cells at 72 h after treated by 5 μM cyclopamine (Fig. 4c).

### Effects of cyclopamine and TMZ in GBM xenografts

**Fig. 5:**
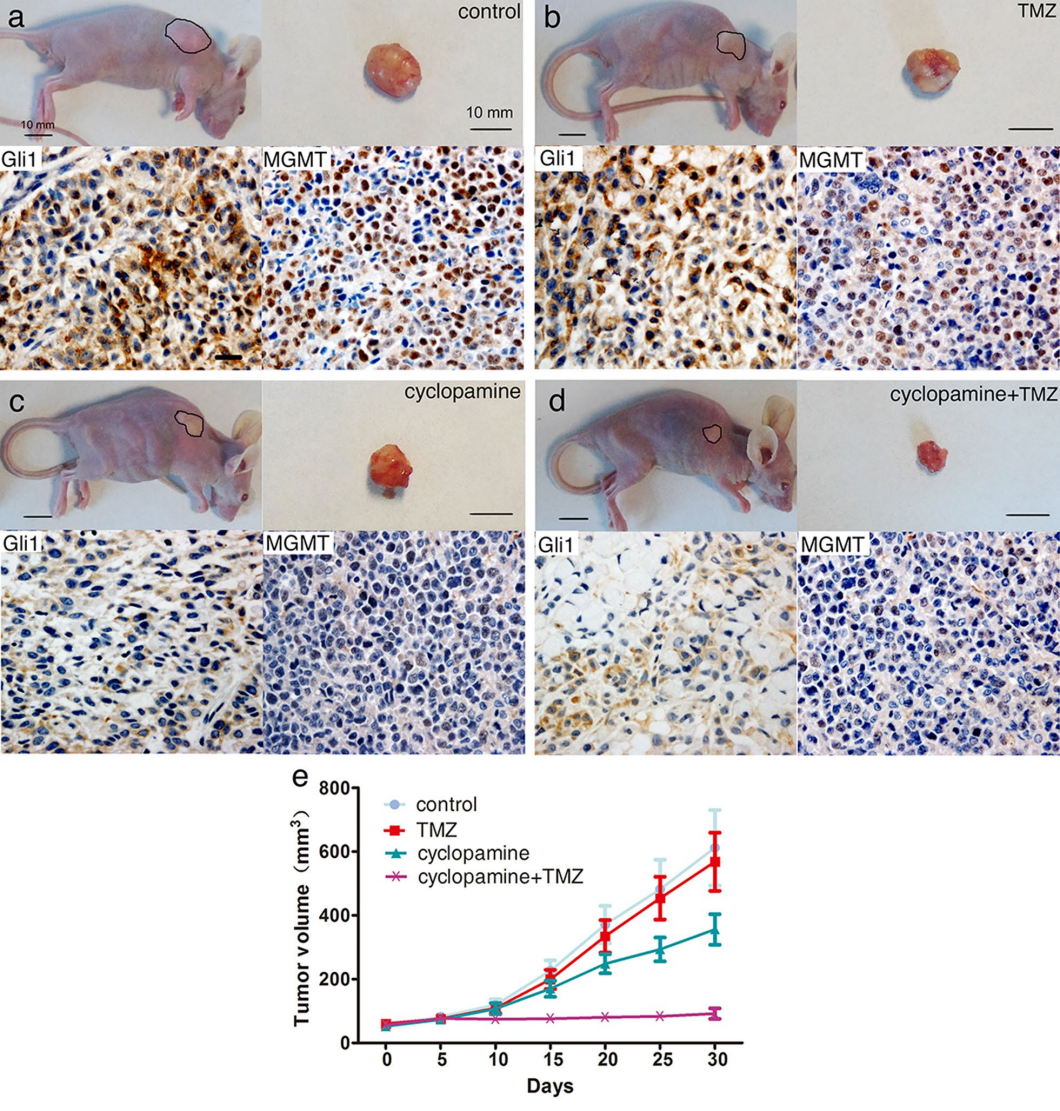
Representative cases of nude mice with GBM xenografts treated with vehicle (**a**), TMZ (**b**), cyclopamine (**c**), or cyclopamine plus TMZ (**d**). Combined TMZ with cyclopamine treatment led to significant tumor growth inhibition in nude mice with GBM xenografts (**e**)

We established GBM xenografts in nude mice using U251 cells to test whether HH/Gli1 signaling activity influenced TMZ sensitivity in in vivo context. It took approximately 2 weeks to reach 5 mm tumor diameter after subcutaneous injection of U251 cells. There were no obvious signs of side effects of the cyclopamine or TMZ, alone or in combination, and no significant body loss in all the four groups of mice. Tumor growth in each group is shown in the Table 1 and Fig. 5e. Statistical analysis showed that the TMZ group had similar tumor size in all days investigated when compared with the control group. The cyclopamine group and the combination group showed significantly smaller tumor size than the control group at 25 and 10 days, respectively. The combination group showed significantly smaller tumor size than the TMZ group and the cyclopamine group at 15 days and thereafter (Table 1). On day 30, when all nude mice were sacrificed, the tumor tissues were obtained and underwent immunohistochemistry for Gli1 and MGMT expression (Fig. 5a–d). The result showed that high levels of Gli1 and MGMT in both the control and TMZ groups while low levels of both the genes were shown in the cyclopamine group and the combination group.

**Table 1:** Tumor growth in the four groups of nude mice

Groups	Tumor volume in 30 days after the treatment by TMZ, cyclopamine, or both (mm^3^)						
	0	5	10	15	20	25	30
Control	54.4 ± 14.7	82.1 ± 27.4	119.1 ± 40.3	226.2 ± 73.5	371.2 ± 131.3	482.1 ± 207.3	612.2 ± 264.9
TMZ	60.4 ± 7.4	75.4 ± 15.5	109.0 ± 35.3	199.3 ± 67.2	334.4 ± 114.0	453.9 ± 149.9	568.3 ± 203.9
Cyclopamine	52.2 ± 7.5	73.5 ± 24.8	107.2 ± 39.0	169.9 ± 57.1	248.5 ± 67.1	293.3 ± 83.6	356.0 ± 106.8
Cyclopamine + TMZ	56.6 ± 7.7	76.8 ± 8.5	74.3 ± 7.0	76.0 ± 10.8	80.5 ± 15.3	83.6 ± 21.8	92.0 ± 37.5
Control vs. TMZ	p = 0.349	p = 0.613	p = 0.639	p = 0.472	p = 0.543	p = 0.746	p = 0.700
Control vs. cyclopamine	p = 0.721	p = 0.514	p = 0.581	p = 0.143	p = 0.055	p = 0.042	p = 0.036
Control vs. cyclopamine + TMZ	p = 0.733	p = 0.687	p = 0.05	p = 0.001	p < 0.001	p < 0.001	p < 0.001
TMZ vs. cyclopamine + TMZ	p = 0.546	p = 0.918	p = 0.119	p = 0.004	p = 0.001	p = 0.001	p = 0.001
Cyclopamine vs. cyclopamine + TMZ	p = 0.487	p = 0.801	p = 0.138	p = 0.02	p = 0.012	p = 0.026	p = 0.031

## Discussion

To date, GBM remains the worst malignant human glioma despite of optimal therapeutic strategy. TMZ is currently recommended as the first-line agent to treat the disease [19]. However, tumor’s resistance to TMZ or tumor recurrence are the main cause of treatment failure. Thus far, few approaches targeting chemoresistance in GBM have been introduced. O^6^-Benzylguanine was used to restore TMZ sensitivity in patients with TMZ-resistant human glioma, but failed to restore TMZ sensitivity in GBM [20]. Interferon-beta inactivates MGMT via p53 gene induction and enhances the therapeutic efficacy to TMZ [21], showing benefits when combined with TMZ to treat newly diagnosed primary GBM [22]. Our study reveals that HH/Gli1 signaling pathway may regulate MGMT expression and chemoresistance to TMZ in GBM.

Nuclear staining of Gli1 is a reliable indicator of HH pathway activity in tumor cells [17]. Here, we investigate the relationship between Gli1 nuclear staining and MGMT expression in primary GMB tissues. The results showed that MGMT-positive tissues have a significantly higher rate of Gli1 nuclear staining than MGMT-negative ones, which implies there is a link between HH/Gli1 signaling activity and MGMT expression. Our following experiments in GBM cell lines revealed the transcriptional regulation of MGMT expression by Gli1 binding to its promoter region, demonstrating MGMT as a downstream target gene of HH/Gli1 signaling pathway. However, it is noteworthy that 5 of 26 (19.2%) MGMT-positive GBM tissues did not show positive nuclear Gli1 staining and 10 of 31 (32.2%) Gli1-positive GBM tissues also did not demonstrate positive MGMT expression. Comparatively, the well-known MGMT promoter methylation status predicts MGMT level more precisely in comparison to our Gli1 staining. We won’t say that Gli1 is a better predictive factor than MGMT promoter methylation status, but as our work shows, we can generate the MGMT expression and sensitivity to the chemotherapy by altering the HH/Gli1 pathway independent from the promoter status. There is a high possibility that 22 of 29 (75.9%) MGMT promoter unmethylated patients have an active HH/Gli1 pathway. We can take advantage here from our work, which could be a potential target that can help overcome the unmethylated patients’ resistance to TMZ. Interestingly, Xu et al. [23] recently found that haplotype has an impact on transcription factor binding in the MGMT promoter/enhancer region consequently regulating MGMT expression. This finding may help our work on Gli1 to fit better on evaluating MGMT activity. Future work can explore the relationship between MGMT promoter and HH/Gli1 pathway statement. With the recruitment of MGMT promoter haplotypes analysis and more IDH-mutant patients, we may be able to establish a better evaluation system of MGMT activity and chemoresistance.

Although several studies have shown that HH/Gli1 signaling pathway is involved in regulation of chemosensitivity in a variety of organ neoplasms, including pancreatic cancers, prostate cancer, and breast caners [24–26], few studies have focused on its influence on chemosensitivity in the central nervous system neoplasm. Activating HH signaling by Gli1 overexpression in A172 cells, resulted in synchronous increase of MGMT expression and enhanced chemoresistance to TMZ. Moreover, blocking the pathway by cyclopamine in U251 cells led to synchronous decrease of MGMT and diminished chemoresistance to TMZ. As the main transcriptional activator, Gli1 enhanced a number of downstream target genes of HH signaling pathway [27]. Our ChIP analysis identified the potential binding site of Gli1 protein on the MGMT promoter region. Therefore, our study proved the involvement of HH/Gli1 signaling in the regulation of TMZ chemosensitivity in GBM.

There is a small subpopulation of tumor cells with stem-like phenotypes in human GBM. Expressing high levels of MGMT, these cells have been shown to be involved in chemotherapy resistance and responsible for tumor recurrence [28]. Moreover, previous studies have shown that these stem-like cancer cells harbor aberrantly activated HH/Gli1 signaling pathway [16, 29]. Furthermore, our study in the U87 GSP cells illustrated that inhibition of the HH/Gli1 signaling pathway by cyclopamine reduced MGMT expression. That indicates the existence of regulatory mechanism at the level of stem/progenitor cells and offers a new angle to mitigate chemoresistance in stem-like cancer cells.

O^6^-Methylguanine DNA methyltransferase expression predicts the response to TMZ in GBM cells [30]. TMZ alone did not effectively inhibit tumor growth because the GBM xenografts originated from the U251 cells that had a high MTMT activity. However, the tumor growth was significantly blocked by cyclopamine possibly due to an activated HH/Gli1 signaling in these cancer cells. The xenografts tissues after cyclopamine treatment showed low levels of Gli1 and MGMT measured by immunohistochemistry, indicating effective inhibition of the HH/Gli1 signaling pathway by cyclopamine. This finding is consistent with the previous study that targeted inhibition of HH signaling resulted in tumor growth repression in malignant glioma xenografts with active HH signaling [31]. In the group of nude mice treated by cyclopamine plus TMZ, tumor growth inhibition was significant at 15 days after the treatment when compared with the control group, which was much earlier than the cyclopamine alone group (25 days). Moreover, the combination group showed significantly smaller tumor volumes than both the cyclopamine group and the TMZ group, demonstrating a synergistic effect on tumor growth inhibition.

In summary, this study shows that HH/Gli1 signaling pathway regulates MGMT expression and chemoresistance to TMZ in human GBM independent from MGMT promoter methylation status, which offers a potential target to restore chemosensitivity to TMZ in a fraction of GBM with high MGMT expression. As complex signaling networks regulating MGMT expression exist at different cellular levels including epigenetics, transcription, and posttranscription [32–34], future studies will shed light on new methods to overcome chemoresistance in human GBM.

## Conclusions

Our studies reveal that HH/Gli1 signaling pathway regulates MGMT expression and chemoresistance to TMZ in human GBM. These findings not only improve our understanding of the novel regulation mechanism of MGMT expression but also offer a potential target to restore chemosensitivity to TMZ in a fraction of GBM with high MGMT expression.

## Abbreviations

TMZ: temozolomide
GBM: glioblastoma
MGMT: O^6^-methylguanine DNA methyltransferase
HH: Hedgehog
PTCH: Patched
SMO: smoothened
MSP: methylation-specific PCR
GSP: glioma stem/progenitor
ChIP: Chromatin Immunoprecipitation
